# Supplementary material for: An Electrochemical Immunosensor Based on SPA and rGO-PEI-Ag-Nf for the Detection of Arsanilic Acid
Source: Molecules. 2021 Dec 28;27(1):172. doi: 10.3390/molecules27010172 (PMC8746453; doi:10.3390/molecules27010172)
Supplement: Supplementary file 1 [file molecules-27-00172-s001.zip › molecules-1518257-supplementary.pdf]

Supplementary Information

Synthesis of rGO-PEI and rGO-PEI-Ag nanocomposites

20 mg GO power was dissolved in 20 mL water and ultrasonicated for 60 minutes to obtain a uniformly distributed GO dispersion. Next, 2mL of 2 mg mL^{-1} PEI was added dropwise to the GO solution and stirred vigorously for 5 hours. Then 40 mg NaOH was added and stirred at 90 °C for 24 h. The color of the dispersion changed from yellow-brown to black, signifying that GO was reduced to rGO. The mixture was centrifuged at 12000 rpm for 5 minutes and washed three times with water to remove impurities and excess physical absorption PEI. Finally, the rGO-PEI precipitate was dried overnight at 80°C.

For the preparation of rGO-PEI-Ag, rGO-PEI solution was obtained by adding 5 mL water to the rGO-PEI power and ultrasonicated for 30 minutes, followed by adding glucose (1.5 g). Meanwhile, ammonium aqueous solution (0.55 M) was added dropwise to 3.35 mL $AgNO_3$ (0.12 M) solution under constant stirring until it became clear, then the obtained silver ammonia solution was added to the mixture containing rGO-PEI and stirred for several minutes. Then the mixture was stand for 1.5 h at room temperature. Afterwards, the mixture was centrifuged and washed several times by water and the precipitate was dried in an oven at 80 °C. Finally, the precipitate was ground and dispersed in water and 0.4% Nf-ethanol solution (3:1) to obtain rGO-PEI-Ag-Nf solution.

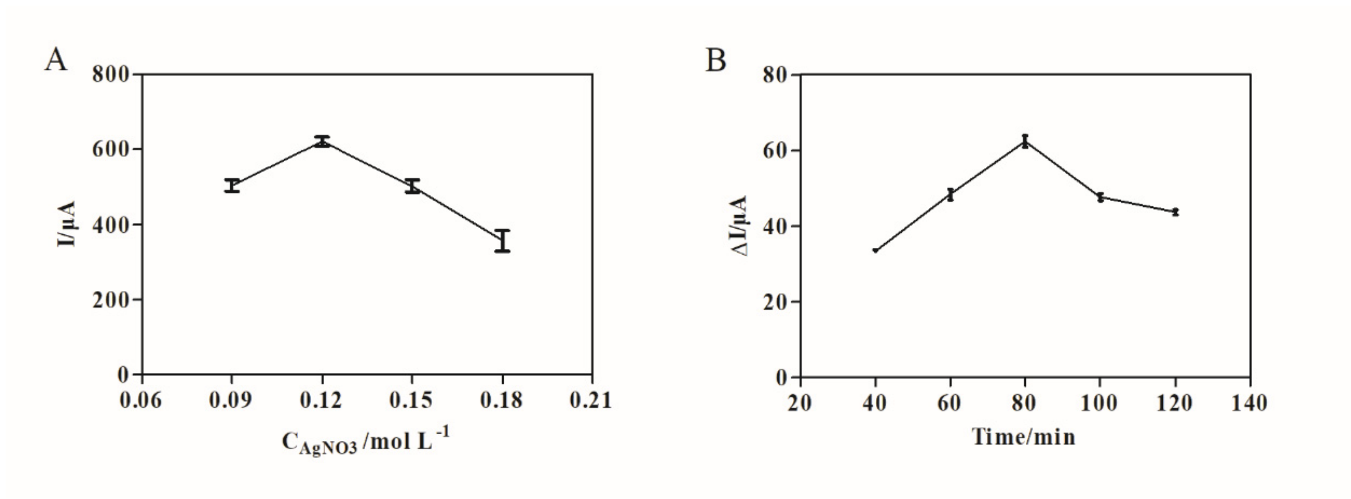

Figure S1 Optimization of concentration of $AgNO_3$ (A) and antigen-antibody binding time (B)

Table S1 Optimization of SPA concentration and incubation time by CVs. (The data in the table was the variation of the intensity of the current (μA).)

Concentration of SPA / Incubation time	0.1 mg mL^{-1}	0.2 mg mL^{-1}	0.3 mg mL^{-1}	0.4 mg mL^{-1}	0.5 mg mL^{-1}
20 minutes	219	205	172	166	256
40 minutes	270	231	183	180	246
60 minutes	265	227	281	250	260
80 minutes	235	221	277	257	263
100 minutes	190	221	265	198	220

Table S2 Optimization of antibody concentration and incubation time by CVs. (The data in the table was the variation of the intensity of the current (μA).)

ASA antibody concentration / Incubation time	1:500	1:1000	1:1500	1:2000
20 minutes	57	50	32	25
40 minutes	78	87	61	40
60 minutes	70	76	67	55
80 minutes	62	59	55	50
100 minutes	52	46	40	34
